# B lymphocyte stimulator (BLyS) isoforms in systemic lupus erythematosus: disease activity correlates better with blood leukocyte BLyS mRNA levels than with plasma BLyS protein levels

**DOI:** 10.1186/ar1855

**Published:** 2005-11-15

**Authors:** Christopher E Collins, Amanda L Gavin, Thi-Sau Migone, David M Hilbert, David Nemazee, William Stohl

**Affiliations:** 1 Division of Rheumatology, Department of Medicine, Los Angeles County + University of Southern California Medical Center, and University of Southern California Keck School of Medicine, 2011 Zonal Avenue, Los Angeles, CA 90033, USA; 2 Department of Immunology, Scripps Research Institute, 10550 North Torrey Pines Road, La Jolla, CA 92037, USA; 3 Human Genome Sciences, Inc., 14200 Shady Grove Road, Rockville, MD 20850, USA

## Abstract

Considerable evidence points to a role for B lymphocyte stimulator (BLyS) overproduction in murine and human systemic lupus erythematosus (SLE). Nevertheless, the correlation between circulating levels of BLyS protein and disease activity in human SLE is modest at best. This may be due to an inadequacy of the former to reflect endogenous BLyS overproduction faithfully, in that steady-state protein levels are affected not just by production rates but also by rates of peripheral utilization and excretion. Increased levels of BLyS mRNA may better reflect increased *in vivo *BLyS production, and therefore they may correlate better with biologic and clinical sequelae of BLyS overexpression than do circulating levels of BLyS protein. Accordingly, we assessed peripheral blood leukocyte levels of BLyS mRNA isoforms (full-length BLyS and ΔBLyS) and plasma BLyS protein levels in patients with SLE, and correlated these levels with laboratory and clinical features. BLyS protein, full-length BLyS mRNA, and ΔBLyS mRNA levels were greater in SLE patients (*n *= 60) than in rheumatoid arthritis patients (*n *= 60) or normal control individuals (*n *= 30). Although full-length BLyS and ΔBLyS mRNA levels correlated significantly with BLyS protein levels in the SLE cohort, BLyS mRNA levels were more closely associated with serum immunoglobulin levels and SLE Disease Activity Index scores than were BLyS protein levels. Moreover, changes in SLE Disease Activity Index scores were more closely associated with changes in BLyS mRNA levels than with changes in BLyS protein levels among the 37 SLE patients from whom repeat blood samples were obtained. Thus, full-length BLyS and ΔBLyS mRNA levels are elevated in SLE and are more closely associated with disease activity than are BLyS protein levels. BLyS mRNA levels may be a helpful biomarker in the clinical monitoring of SLE patients.

## Introduction

B lymphocyte stimulator (BLyS; a trademark of Human Genome Sciences, Inc., Rockville, MD, USA) is a 285-amino-acid member of the tumor necrosis factor ligand superfamily [1-3]. A causal relation between constitutive overproduction of BLyS and development of systemic lupus erythematosus (SLE)-like illness has incontrovertibly been established in mice. BLyS-transgenic mice often develop SLE-like features as they age [3-5], and SLE-prone (NZB × NZW)F_1 _(BWF_1_) and MRL-*lpr/lpr *mice respond clinically to treatment with BLyS antagonists (decreased disease progression and improved survival) [3,6].

Considerable inferential evidence points to a role for BLyS overproduction in human SLE as well. Cross-sectional studies have demonstrated elevated circulating levels of BLyS in 20–30% of human SLE patients tested at a single point in time [7,8]. Moreover, a 12-month longitudinal study documented persistently elevated serum BLyS levels in about 25% of SLE patients and intermittently elevated serum BLyS levels in an additional 25% of patients [9]. Remarkably, circulating BLyS levels did not correlate with disease activity (measured using the SLE Disease Activity Index [SLEDAI]) in these cross-sectional or longitudinal studies [7-9]. Although a statistically significant correlation between circulating BLyS levels and SLEDAI has been appreciated in a more recent 24-month longitudinal study of 245 SLE patients (with >1,700 plasma samples analyzed) [10], the correlation remains weak.

The limited correlation between circulating BLyS protein levels and disease activity in these studies may have exposed an inadequacy of the former to reflect faithfully endogenous BLyS overproduction. In addition to the rate of BLyS protein production, several other factors (for example, utilization and excretion) can affect circulating BLyS protein levels. Although there are no practicable means of directly measuring *in vivo *BLyS production *per se *in humans, the level of BLyS mRNA may serve as a better surrogate marker of *in vivo *BLyS production than does the level of BLyS protein. Candidate BLyS mRNA isoforms include the full-length BLyS mRNA isoform, which encodes the full-length protein, and the alternatively spliced ΔBLyS mRNA isoform, which encodes a protein with a small peptide deletion [11]. (ΔBLyS does not bind to cells expressing BLyS receptors, and therefore it has no agonistic activity. Moreover, ΔBLyS can form heterotrimers with full-length BLyS, thereby actually functioning as a dominant-negative antagonist of BLyS activity.)

In this report we demonstrate that peripheral blood leukocytes from SLE patients express elevated mRNA levels of both full-length BLyS and ΔBLyS relative to those levels expressed by patients with rheumatoid arthritis (RA) or by normal control individuals. In the SLE patients, both full-length BLyS and ΔBLyS mRNA levels are more closely associated with disease activity (SLEDAI) than are BLyS protein levels. Accordingly, BLyS mRNA levels may be a helpful biomarker in the clinical monitoring of SLE patients.

## Materials and methods

### General details

This study was approved by the institutional review boards of the University of Southern California and the Scripps Research Institute. All participants gave their written informed consent before participation in this study.

### Participants

Patients receiving outpatient medical care at the rheumatology clinics of the Los Angeles County + University of Southern California Medical Center were recruited into the study. Diagnoses of SLE (*n *= 60) or RA (*n *= 60) were based on established clinical criteria [12]. Healthy control individuals (*n *= 30) were recruited from Los Angeles County + University of Southern California Medical Center and University of Southern California Keck School of Medicine personnel. No exclusions were made on any basis other than an inability to give informed consent. Each patient's sex, race, age, and medications at the time of the phlebotomy were recorded (Table 1).

Based solely on the patient's willingness to donate a second blood sample, repeat blood samples were collected from 37 of the SLE patients 147–511 days (median 371 days) after collection of the first samples. These patients were not selected on the basis of any demographic, clinical, or laboratory feature.

Clinical disease activity for the SLE patients was assessed using the SLEDAI [13] and using a modified SLEDAI that excludes the contribution of anti-double-stranded DNA (anti-dsDNA) antibodies from the total score. Each patient's medical chart was reviewed for results of standard clinical laboratory tests within the previous or subsequent 1-month period.

### Plasma BLyS determination

Whole venous blood was centrifuged to yield plasma and a buffy coat. The plasma was harvested, stored at -70°C, and assayed for BLyS levels by ELISA [8,14] using Fab fragments of the capture antibody rather than the whole antibody to reduce assay interference by rheumatoid factor. The lower limit of detection in this assay is 0.3 ng/ml. For statistical purposes, plasma samples with BLyS concentrations below the lower limit of detection were assigned a value of 0.25 ng/ml.

### Blood BLyS mRNA determination

The buffy coat from centrifuged whole blood was harvested, added to RNAlater™ (Ambion, Austin, TX, USA) at a 1:4 vol/vol ratio for RNA stabilization, stored at -70°C, and assayed for full-length BLyS and ΔBLyS mRNA levels by real-time PCR. Total RNA was purified from buffy coat samples using RNAeasy miniprep kits (Qiagen, Valencia, CA, USA), and contaminating genomic DNA was removed by DNAse-I digestion. One-tenth volume of total RNA was used as template in the first-strand cDNA reaction using oligo-dT and the Superscript III first-strand synthesis system (Invitrogen, Carlsbad, CA, USA). Duplicate samples of cDNA were amplified with primers against β-actin, full-length BLyS, or ΔBLyS: β-actin sense 5'-CGAGAAGATGACCCAGATCATGT-3'; β-actin anti-sense 5'-GGCATACCCCTCGTAGATGG-3'; full-length BLyS sense 5'-GCAGACAGTGAAACACCAACTATAC-3'; ΔBLyS sense 5'-CAGAAGAAACAGGATCTTACACAT-3'; and full-length BLyS/ΔBLyS anti-sense 5'-TGCCAGCTGAATAGCAGGAATTAT-3'.

A 165 bp amplicon for β-actin was PCR-amplified using the 7900 HT ABI Prism machine (Qiagen) with annealing at 65°C. A 296 bp amplicon for full-length BLyS was PCR-amplified, with annealing at 64°C. A 270 bp amplicon for ΔBLyS was PCR-amplified with annealing at 61°C. The annealing conditions for full-length BLyS and ΔBLyS were determined so that each primer set remained specific to the respective BLyS isoform and yielded a PCR efficiency similar to those of cloned cDNA standards. Melting curve analysis revealed a single peak for each gene amplified. The threshold cycle (Ct) values for each reaction were determined using Sequence Detection System software (Applied Biosystems, Foster City, CA, USA). Results are presented as ratios of full-length BLyS or ΔBLyS mRNA to β-actin mRNA, which were calculated using the following formulae:

2 exp(Ct_β-actin _- Ct_full-length BLyS_)

2 exp(Ct_β-actin _- Ct_ΔBLyS_)

### Determination of anti-BLyS autoantibodies

BLyS was bound to microtiter plates by first coating the plates with streptavidin and then adding biotinylated recombinant BLyS. Using these plates as the capture reagent, plasma samples were incubated, and horseradish peroxidase-conjugated anti-human IgA/IgM/IgG (Southern Biotechnology Associates, Birmingham, AL, USA; 1:20,000 final dilution) or horseradish peroxidase-conjugated anti-human IgG (Southern Biotechnology; 1:10,000 final dilution) were used as the detector reagents.

### Statistical analysis

All analyses were performed using SigmaStat software (SPSS, Chicago, IL, USA). Results that did not follow a normal distribution were log-transformed to achieve normality. Parametric testing between two matched or unmatched groups was performed using the paired or unpaired *t* test, respectively. Parametric testing among three or more groups was performed using one-way analysis of variance. When log-transformation failed to generate normally distributed data or the equal variance test was not satisfied, nonparametric testing was performed using the Mann–Whitney rank sum test between two groups and by Kruskal–Wallis one-way analysis of variance on ranks among three or more groups. Correlations were determined using Pearson product moment correlation for interval data and using Spearman rank order correlation for ordinal data or for interval data that did not follow a normal distribution. Nominal data were analyzed using χ^2 ^analysis-of-contingency tables.

## Results

### Elevated plasma BLyS levels and blood levels of full-length BLyS and ΔBLyS mRNA isoforms in systemic lupus erythematosus patients

**Figure 1:**
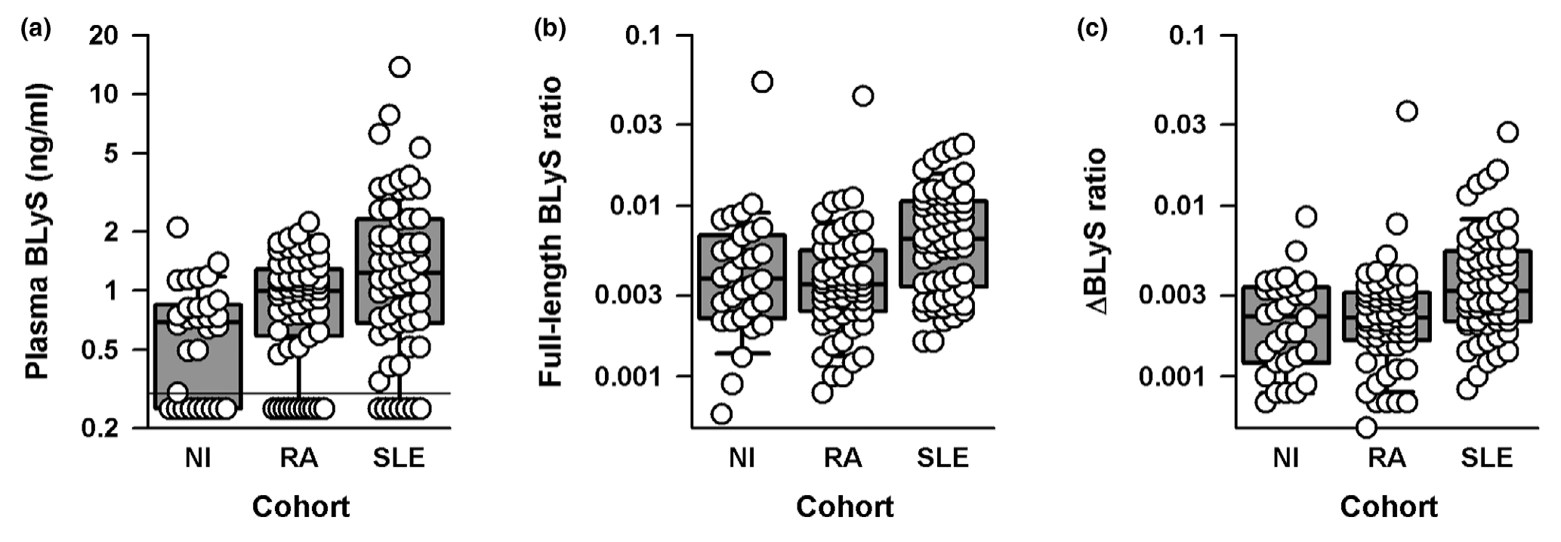
BLyS protein and BLyS isoform mRNA levels in normal individuals, and RA, and SLE patients. **(a) **Plasma from normal individuals (Nl), and RA and SLE patients were assayed for BLyS levels by ELISA. Each symbol indicates an individual subject. The composite results are plotted as box plots. The lines inside the boxes indicate the medians, the outer borders of the boxes indicate the 25th and 75th percentiles, and the bars extending from the boxes indicate the 10th and 90th percentiles. The thin solid line across the plot indicates the lower limit of detection in the BLyS assay (0.3 ng/ml). **(b,c) **RNA was extracted from peripheral blood buffy coats from the same individuals. Amounts of full-length BLyS, ΔBLyS, and actin mRNA were quantified by real-time RT-PCR, and results are shown as ratios of full-length BLyS mRNA (b) and ΔBLyS mRNA (c) to actin mRNA. BLyS, B lymphocyte stimulator; ELISA, enzyme-linked immunosorbent assay; RA, rheumatoid arthritis; RT-PCR, reverse transcription polymerase chain reaction; SLE, systemic lupus erythematosus.

Previous reports of elevated circulating BLyS levels in SLE patients were based on a BLyS ELISA that utilized a whole (unfragmented) capture anti-BLyS monoclonal antibody [7-9]. Since the publication of these reports, it has been recognized that the presence of rheumatoid factor can potentially interfere with the assay and lead to spurious overestimation of the true circulating BLyS levels (Human Genome Sciences, Inc.; unpublished observations). To mitigate potential interference from rheumatoid factor, the BLyS ELISA has been modified and the capture anti-BLyS monoclonal antibody is now utilized as a Fab fragment. Despite the changes in the ELISA format, our findings are entirely consistent with those of the previous reports. Plasma BLyS levels were significantly greater in the SLE group than in either RA or normal control group (*P *< 0.001; Figure 1a). Arbitrary assignment of the 95th percentile value among the normal control individuals as the upper limit of 'normal' revealed that two of the 30 normal control individuals, 15 of the 60 RA patients, and 29 of the 60 SLE patients harbored elevated plasma BLyS levels (*P *< 0.001).

Overexpression of BLyS in SLE patients was also established by measuring BLyS mRNA levels normalized to β-actin mRNA levels in peripheral blood leukocytes (buffy coats). The geometric mean full-length BLyS mRNA and ΔBLyS mRNA levels among the SLE patients were each significantly greater than those among the RA patients and normal control individuals, respectively (*P *< 0.001 for each; Figure 1b,c). Arbitrary assignment of the 95th percentile values for full-length BLyS and ΔBLyS mRNA levels among the normal control individuals as the upper limits of 'normal' revealed that two of the 30 normal control individuals, four of the 60 RA patients, and 20 of the 60 SLE patients had elevated full-length BLyS mRNA levels (*P *< 0.001), and that two of the 30 normal control individuals, three of the 60 RA patients, and 19 of the 60 SLE patients had elevated ΔBLyS mRNA levels (*P *< 0.001). Levels of full-length BLyS and ΔBLyS mRNA strongly correlated with each other (r = 0.703; *P *< 0.001) in the SLE cohort, and plasma BLyS levels also correlated significantly with levels of each BLyS isoform (r = 0.429, *P *< 0.001; and r = 0.290, *P *= 0.024, respectively). Among these SLE patients, none of the measured BLyS parameters correlated with patient age, sex, race, or daily dose of corticosteroids (data not shown). Because the racial composition of the normal cohort was not as predominantly Hispanic as were those of the RA and SLE cohorts, we assessed the BLyS parameters in the respective Hispanic subpopulations. As for the entire populations, values for SLE were significantly greater than those for either RA or normal controls (*P *≤ 0.004; data not shown).

### Correlations between BLyS parameters and plasma immunoglobulin levels

**Figure 2:**
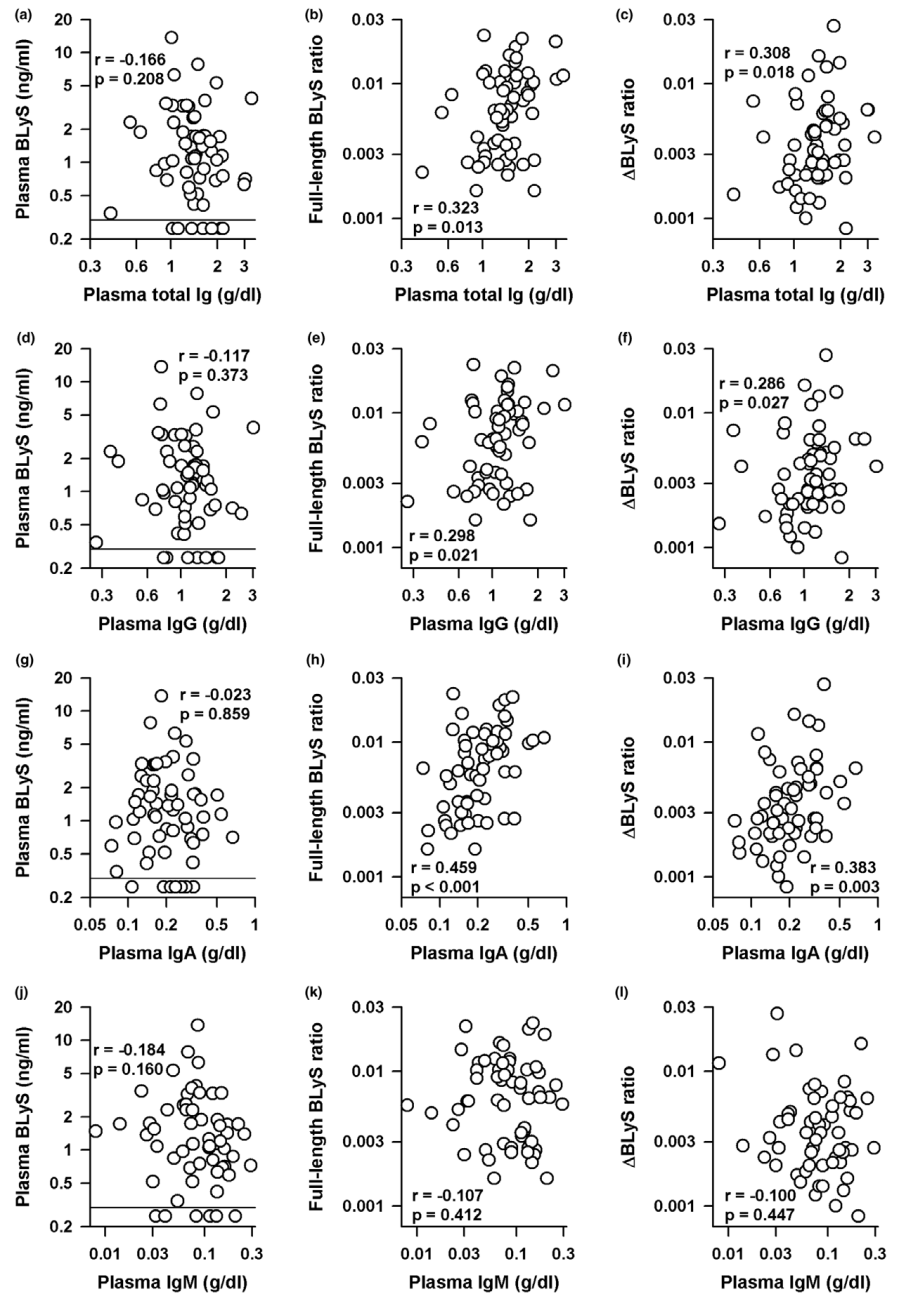
Correlations in the SLE patients between BLyS parameters and plasma immunoglobulin levels. Individual **(a,d,g,j) **BLyS protein levels, **(b,e,h,k) **full-length BLyS mRNA levels, and **(c,f,i,l) **ΔBLyS mRNA levels are plotted against corresponding plasma total immunoglobulin levels (a–c), IgG levels (d–f), IgA levels (g–i), and IgM levels (j–l). BLyS, B lymphocyte stimulator; IL, interleukin.

BLyS is a potent B cell survival factor [15-21], and administration of exogenous BLyS to mice leads to B cell expansion and hypergammaglobulinemia [1]. Previous studies with numbers of SLE patients greater than were included in the present study documented a modest but significant correlation between serum levels of BLyS and IgG [8,10]. In our SLE cohort of limited size, plasma BLyS levels failed to show significant correlations with plasma levels of total immunoglobulin, IgG, or IgA. In contrast, full-length BLyS and ΔBLyS mRNA levels correlated significantly with each (Figure 2). (None of the BLyS parameters correlated with plasma IgM levels.) The absence of significant correlation between plasma BLyS levels and the immunoglobulin parameters also persisted when just the 53 patients with detectable plasma BLyS levels were considered (r = -0.133, *P *= 0.346 for total immunoglobulin; r = -0.048, *P *= 0.734 for IgG; and r = 0.033, *P *= 0.817 for IgA).

### Correlations between BLyS parameters and disease activity

**Figure 3:**
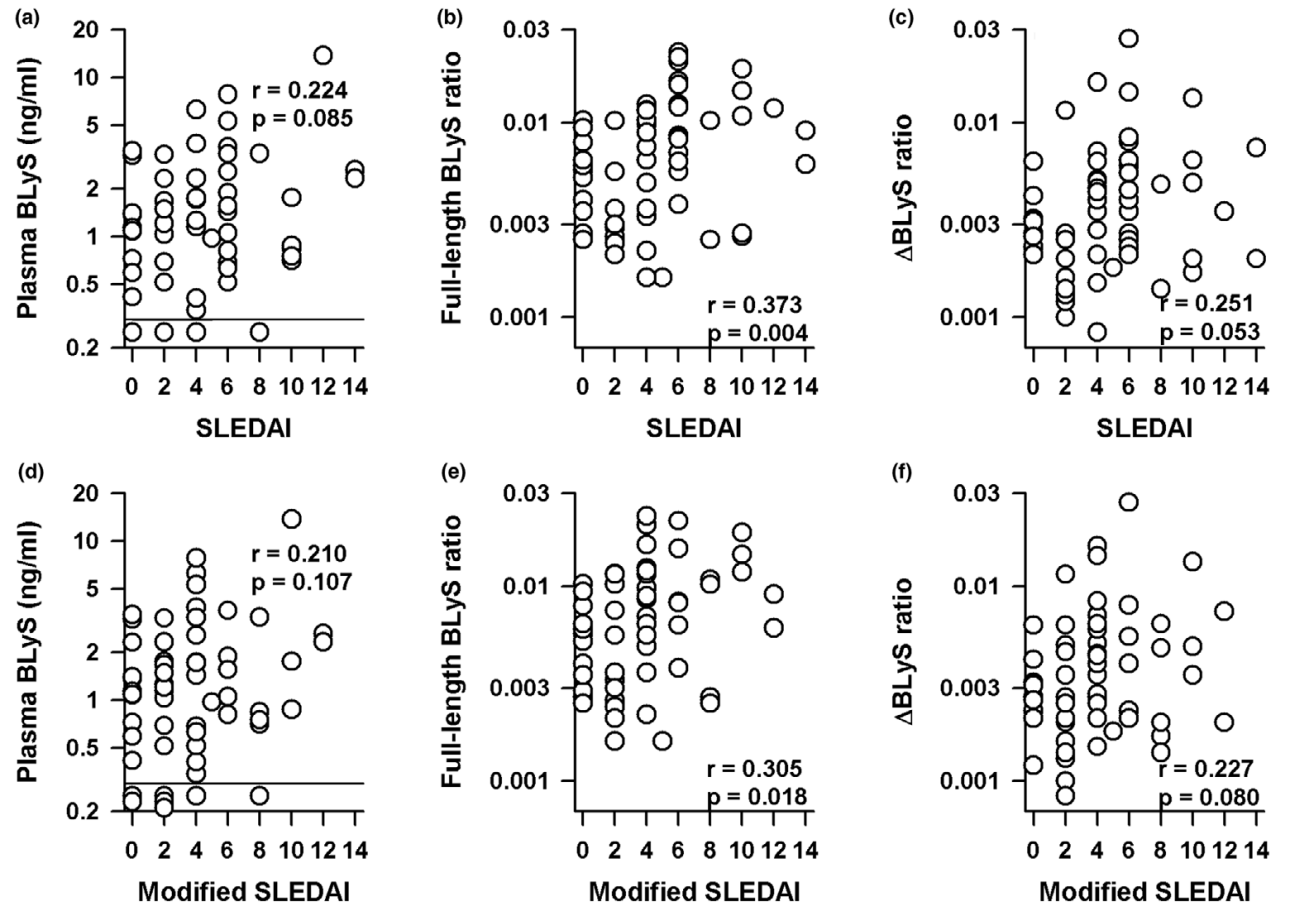
Correlations in the SLE patients between BLyS parameters and disease activity. Individual **(a,d) **BLyS protein levels, **(b,e) **full-length BLyS mRNA levels, and **(c,f) **ΔBLyS mRNA levels are plotted against corresponding SLEDAI scores (a–c) or modified SLEDAI scores (d–f). BLyS, B lymphocyte stimulator; SLEDAI, Systemic Lupus Erythematosus Disease Activity Index.

Previous studies either have failed to demonstrate a significant correlation between disease activity and circulating BLyS levels [7-9] or have detected only a weak correlation between the two [10]. Consonant with those studies, we identified no significant correlation between plasma BLyS levels and SLEDAI in our cohort of 60 SLE patients (Figure 3a). The failure to demonstrate a significant correlation cannot be attributed to a skewing of the results by the patients in whom plasma BLyS levels were below the limit of detection, because no significant correlation was detected among the 53 SLE patients in whom plasma BLyS levels were in the detectable range (r = 0.185, *P *= 0.183). In contrast, a significant correlation between SLEDAI and full-length BLyS mRNA levels was readily discernible (Figure 3b). A trend toward a correlation between SLEDAI and ΔBLyS mRNA levels was also observed, although it did not achieve statistical significance (Figure 3c).

A component of the SLEDAI is the presence of circulating anti-dsDNA antibodies. Because circulating BLyS levels may affect the presence and/or titers of circulating anti-dsDNA antibodies [7-10], we assessed correlations between the individual BLyS parameters and a modified SLEDAI that excludes any consideration of anti-dsDNA antibodies. As with the unmodified SLEDAI, the modified SLEDAI did not correlate with plasma BLyS levels (Figure 3d) either among the SLE cohort overall or among the 53 patients in whom plasma BLyS levels were in the detectable range (r = 0.160, *P *= 0.252), but it significantly correlated with full-length BLyS mRNA levels (Figure 3e) and exhibited a trend toward correlation with ΔBLyS mRNA levels (Figure 3f). Thus, the stronger correlations between BLyS mRNA levels and disease activity cannot solely be explained by any effects that BLyS may have on anti-dsDNA antibodies *per se*.

**Figure 4:**
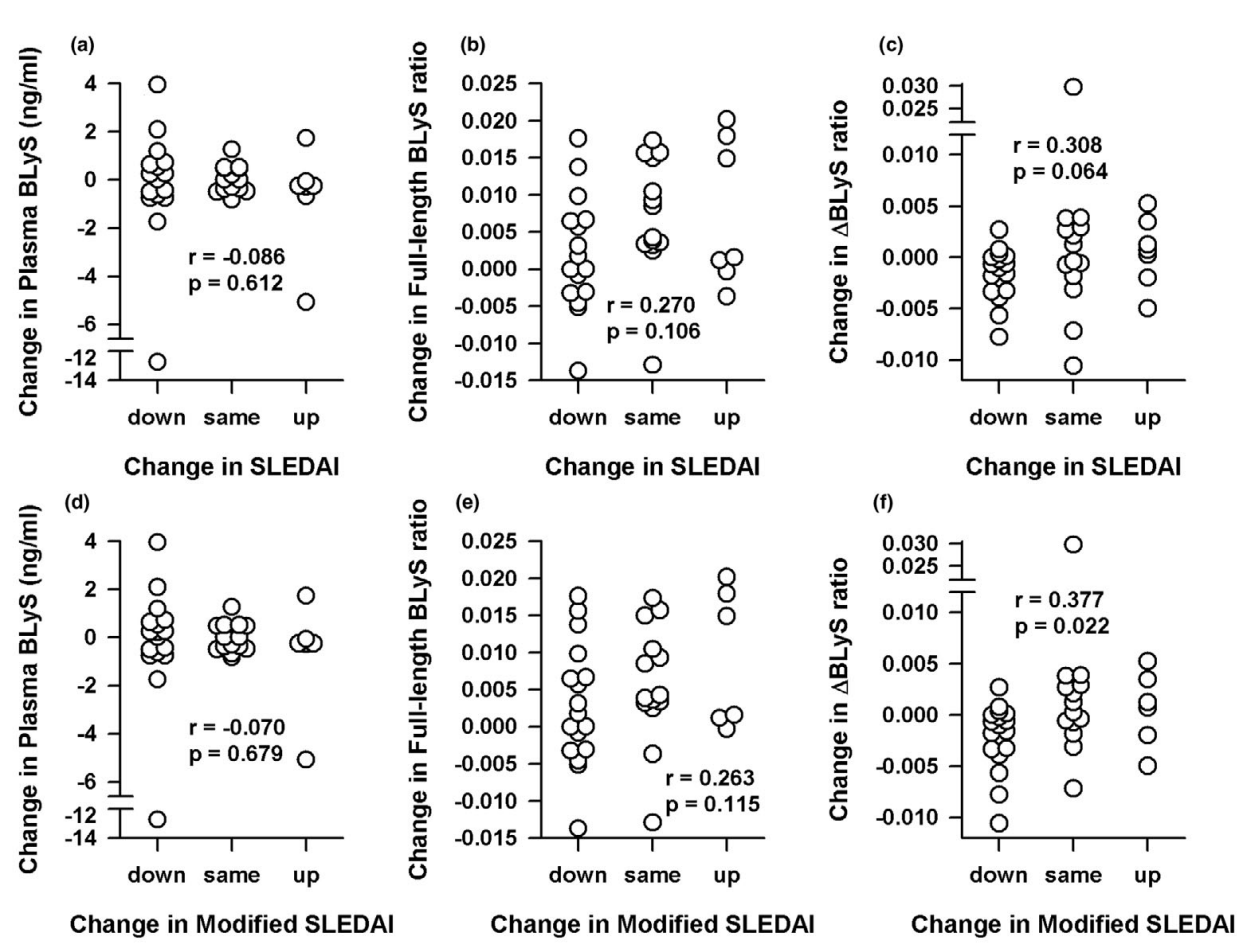
Correlations in SLE patients between changes in SLEDAI and BLyS parameters. Individual **(a,d) **BLyS protein levels, **(b,e) **full-length BLyS mRNA levels, and **(c,f) **ΔBLyS mRNA levels are plotted according to directional changes in SLEDAI scores (a–c) or modified SLEDAI scores (d–f) between the first and second clinical assessments. BLyS, B lymphocyte stimulator; SLEDAI, Systemic Lupus Erythematosus Disease Activity Index.

Moreover, among the 37 SLE patients who were evaluated on two separate occasions, trends toward correlation were appreciated between changes in the unmodified or modified SLEDAI and changes in full-length BLyS or ΔBLyS mRNA levels but not changes in plasma BLyS levels (Figure 4). These results cannot be ascribed to changes in medications taken by the patients, because changes in neither disease activity nor in any of the BLyS parameters correlated with changes in the doses of corticosteroids or cytotoxics taken by the patients (data not shown). The failure to demonstrate a meaningful association between changes in SLEDAI score and changes in plasma BLyS protein levels cannot be attributed to a skewing of the results by the patients in whom plasma BLyS levels were below the limit of detection, because the absence of association between the two persisted among the 27 SLE patients in whom plasma BLyS levels were in the detectable range in both samples (r = -0.069, *P *= 0.727 for plasma BLyS versus unmodified SLEDAI; r = -0.020, *P *= 0.919 for plasma BLyS versus modified SLEDAI).

### Lack of correlation between levels of BLyS mRNA isoforms and percentages of individual leukocyte cell types

**Figure 5:**
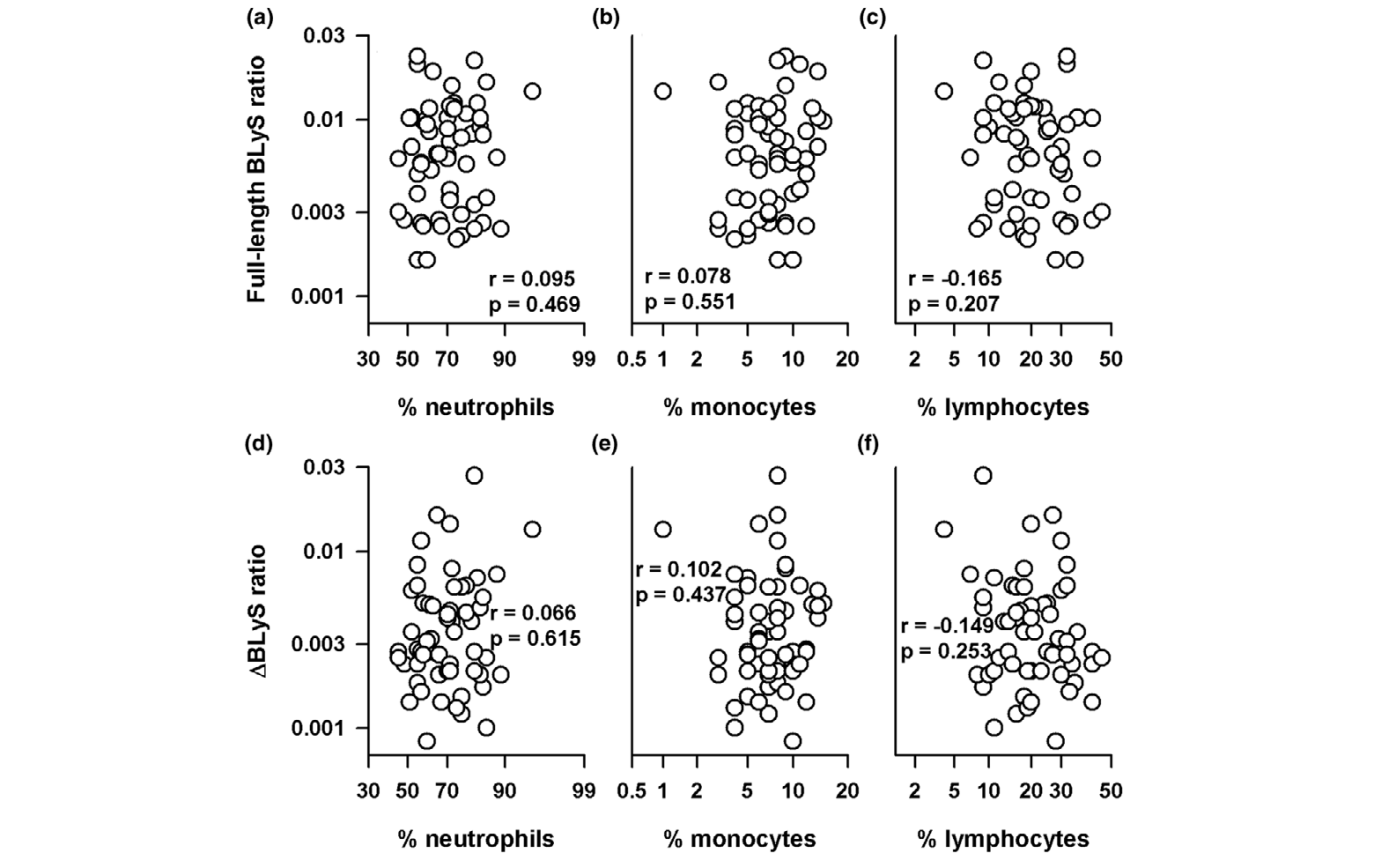
No correlation in SLE between BLyS mRNA isoforms and percentages of individual leukocyte cell types. Individual **(a–c) **full-length BLyS mRNA levels and **(d–f) **ΔBLyS mRNA levels are plotted against corresponding peripheral blood percentages of neutrophils (a,d), monocytes (b,e), and lymphocytes (c,f). BLyS, B lymphocyte stimulator.

Among cells in peripheral blood, BLyS is predominantly expressed by cells of the myeloid lineage (monocytes and neutrophils) [1,14,22,23]. Accordingly, a shift in the differential leukocyte count away from lymphocytes to monocytes and/or neutrophils could substantially alter BLyS mRNA results. Because of the limited amount of blood we were permitted to obtain from the SLE patients (consequent to the high prevalence of anemia among these patients), we were unable to purify the individual leukocyte populations for BLyS mRNA analysis. Nevertheless, to demonstrate that the elevated BLyS mRNA levels in SLE did not simply reflect a shift in differential leukocyte count, we assessed the correlations between the individual BLyS parameters on the one hand and the percentages of blood neutrophils, monocytes, and lymphocytes on the other. No correlations were appreciated (Figure 5).

### Presence of anti-BLyS autoantibodies in patients with systemic lupus erythematosus

The poorer correlation between plasma BLyS protein levels and disease activity compared with that between BLyS mRNA levels and disease activity was striking. Patients with SLE frequently develop autoantibodies against self-antigens, and so some of the SLE patients might have harbored autoantibodies to BLyS. Such autoantibodies could have complexed with BLyS and enhanced its clearance, thereby masking BLyS overproduction. Alternatively, such autoantibodies might have sterically blocked the epitopes recognized by the detecting antibodies in the *in vitro *ELISA. In this case, measured BLyS levels would have been spuriously reduced, again masking BLyS overproduction.

In our cohort, IgA/IgM/IgG anti-BLyS antibodies were detected in six out of the 60 SLE patients. Such autoantibodies were also detected in two out of 60 RA patients and in one out of 30 normal control individuals, demonstrating that anti-BLyS autoantibodies are not restricted to SLE patients. IgG anti-BLyS autoantibodies were detected in 3 SLE patients but in no RA patients or normal control individuals.

## Discussion

Elevated blood levels of BLyS protein and mRNA are well described features of human SLE [7-9]. We confirmed these observations in our study and extended them by documenting increases not just in levels of full-length BLyS mRNA but also in levels of ΔBLyS mRNA (Figure 1). Of note, BLyS mRNA levels were elevated in SLE but not in RA, raising the possibility that BLyS overproduction in SLE is systemic whereas BLyS overproduction in RA may be more focused to the affected arthritic joints [24]. The modestly elevated plasma BLyS protein levels in RA patients may reflect, at least in part, release of locally overproduced BLyS into the circulation.

The relationship between circulating BLyS protein levels and disease activity was addressed in several previous studies, but significant correlations between the two measures did not emerge [7-9]. In the largest study to date, a 2-year longitudinal study of 245 patients in which more than 1,700 plasma samples were analyzed, a significant but weak correlation between the two was appreciated [10]. In the present study, a significant correlation between plasma BLyS protein levels and disease activity was again not realized (Figure 3a,d).

The weak, at best, correlation between circulating BLyS levels and disease activity is seemingly rather surprising. There is a clear-cut association in BlyS transgenic mice between BLyS overexpression and development of SLE-like features [3-5], and treatment of SLE-prone mice with BLyS antagonists retards the progression of disease and improves survival [3,6]. Moreover, development of precocious glomerular pathology in autoimmune-prone mice correlates strongly with circulating BLyS levels [25].

The likely explanation for the weak correlation between circulating BLyS levels and disease activity in human SLE is not that disease activity in SLE patients is insensitive to the degree of BLyS overproduction. Rather, a more tenable explanation is that circulating BLyS levels in human SLE do not always accurately reflect excessive endogenous BLyS production. We can identify at least three nonmutually exclusive mechanisms to explain a dissociation between the two.

First, SLE patients frequently develop autoantibodies to a myriad of self-targets (for example, erythrocytes, lymphocytes). Indeed, we detected circulating IgA/IgM/IgG anti-BLyS autoantibodies in 10% (6/60) of the tested SLE patients, and we detected circulating IgG anti-BLyS autoantibodies in 5% (3/60). These percentages may be underestimates of the true prevalence of anti-BLyS autoantibodies, because some of these autoantibodies may be saturated *in vivo *with circulating BLyS, rendering them incapable of binding to BLyS in the *in vitro *detection assay. We do not yet know whether the anti-BLyS autoantibodies are functionally neutralizing but, regardless, such autoantibodies could enhance the clearance of BLyS and/or interfere with *in vitro *detection of BLyS, thereby masking endogenous BLyS overproduction.

Second, increased urinary excretion of BLyS has been reported in SLE patients, especially among those with clinically overt renal involvement [26]. At least four of the patients we studied manifested nephrotic-range proteinuria (≥3 g/24 hours), and so urinary loss of BLyS was probably substantial in at least these patients. A validated assay for urinary BLyS detection has not yet been developed so we were unable to quantify urinary BLyS levels. Once an assay for urinary BLyS levels is validated, we should be able to assess the effect of urinary BLyS excretion on circulating BLyS levels.

Third, BLyS promotes *in vivo *expansion of B cells [1]. Freshly isolated SLE B cells, despite intact surface expression of BLyS receptors, bind less biotinylated BLyS *ex vivo *than do freshly isolated normal B cells [27]. Although other interpretations are possible, the most likely explanation is that BLyS receptors on B cells in SLE patients are occupied *in vivo *by soluble BLyS. Accordingly, it is likely that BLyS receptors expressed by the expanded B cell population do bind BLyS and remove it from the circulation, resulting in a homeostatic pathway that modulates the effects of BLyS overproduction on circulating BLyS levels. Indeed, circulating levels of BLyS rise with peripheral blood B cell depletion and fall with re-emergence of peripheral blood B cells in rituximab-treated RA or SLE patients [28,29], highlighting this inverse relationship between circulating BLyS levels and B cell load. Moreover, one of the hallmarks of active disease in human SLE is the increased percentages of activated B cells and plasma cells in peripheral blood [30-34], probably reflecting increased systemic numbers of activated B cells and plasma cells. Although not yet formally tested, differential BLyS receptor expression by these cells compared with expression by nonactivated B cells may result in increased peripheral BLyS utilization, further dampening the effects of BLyS overproduction on circulating protein levels.

To circumvent these confounding processes, we used BLyS mRNA levels as a surrogate marker of endogenous BLyS production. Overall, the correlations between disease activity and either full-length BLyS or ΔBLyS mRNA levels were much stronger than that between disease activity and BLyS protein levels (Figures 3 and 4). This was the case regardless of whether we used the standard SLEDAI or the modified SLEDAI as a measure of disease activity. These correlations were not spurious ones consequent to shifts in percentages of leukocyte subpopulations in peripheral blood, because BLyS mRNA levels did not correlate with percentages of blood neutrophils, monocytes, or lymphocytes (Figure 5).

A similar pattern was observed between plasma immunoglobulin levels and the BLyS parameters, with plasma levels of total immunoglobulin, IgG, and IgA correlating significantly with full-length BLyS and ΔBLyS mRNA levels but not with plasma BLyS levels (Figure 2). These significant correlations between full-length BLyS or ΔBLyS mRNA levels and plasma immunoglobulin levels again highlight the greater ability of BLyS mRNA levels, compared with plasma BLyS protein levels, to reflect ongoing BLyS overproduction.

At present, it is not known whether soluble ΔBLyS protein is present in the circulation of SLE patients or of normal individuals. Although full-length BLyS protein is readily cleaved and released from cells transfected with a vector containing murine full-length BLyS, ΔBLyS protein is not cleaved or released from murine ΔBLyS transfectants [11]. Given the strong similarities between murine and human full-length BLyS and ΔBLyS, it is likely that human soluble ΔBLyS protein is also not cleaved from the cell surface and released into the circulation. Moreover, soluble ΔBLyS protein is not released from cells transfected with a vector containing just the extracellular domain of human ΔBLyS (which encodes the soluble protein; A.L. Gavin, unpublished observations). Whether this reflects rapid intracellular degradation of soluble ΔBLyS or some other impediment to its release remains unknown. Regardless, if the inability to release soluble ΔBLyS *in vitro *faithfully recapitulates *in vivo *biology, then the stronger associations between SLE disease activity and full-length BLyS or ΔBLyS mRNA levels compared with that between SLE disease activity and BLyS protein levels could not be attributable to interference by biologically inactive (inhibitory) ΔBLyS protein in the BLyS protein detection ELISA. Importantly, even if soluble ΔBLyS protein is present in the circulation and is detected by the BLyS protein detection ELISA, then the stronger correlations between SLE disease activity and full-length BLyS or ΔBLyS mRNA levels than that between disease activity and total BLyS (including ΔBLyS) protein levels suggest that full-length BLyS and/or ΔBLyS mRNA levels may operationally serve as useful biomarkers of disease activity in SLE. Although the complexity and labor intensiveness associated with quantitative real-time PCR may render measurement of BLyS mRNA levels impracticable for routine clinical practice, such measurement could readily be incorporated into clinical trials and yield valuable information.

Longitudinal observations in large numbers of SLE patients will be necessary to establish or refute the utility of full-length BLyS and/or ΔBLyS mRNA to subserve this clinically vital function.

Although expression of the two major BLyS isoforms was highly coordinate among SLE patients, there were several patients in whom ΔBLyS mRNA levels were markedly greater than or less than the expected values based on full-length BLyS mRNA levels (data not shown). This raises the possibility that dysregulation of ΔBLyS may contribute to overall BLyS dysregulation in at least some SLE patients. It is known that interferon-γ, interleukin-10, interferon-α, and CD154 can upregulate full-length BLyS mRNA levels [14,22,35], but it is not known what effects these or other cytokines/cell-surface structures have on ΔBLyS expression. Further investigation of the regulation of ΔBLyS and the differential expression of BLyS isoforms is certainly warranted.

Although the associations between full-length BLyS and/or ΔBLyS mRNA levels and disease activity in SLE were usually strong when the SLE cohort was analyzed in aggregate, there were several SLE patients in whom BLyS mRNA levels were quite high despite little objective ongoing disease activity, and there were several SLE patients in whom BLyS mRNA levels were low despite considerable ongoing disease activity. One must recognize that the bulk of the pathogenic autoimmune responses probably takes place in the spleen and lymph nodes, rather than in the peripheral blood, where myeloid lineage cells (for example, dendritic cells) produce BLyS and support B cell survival and expansion [36]. Local BLyS production in the secondary lymphoid tissues will be more important to the development and maintenance of an autoimmune response than will remote BLyS levels in the circulation. Because at least some autoreactive B cells may be more sensitive to BLyS-mediated survival signals than non-autoreactive B cells [37,38], local increases in BLyS production could preferentially promote expansion of autoreactive B cells. These cells, in turn, could activate autoreactive T cells by presenting autoantigen to them, and some of the autoreactive B cells would respond to T cell derived signals and mature into (pathogenic) autoantibody secreting plasma cells. In contrast to murine studies, in which investigators can readily harvest and analyze lymphoid and myeloid lineage cells from any site (for example, spleen, bone marrow), such is not the case for human studies. Peripheral blood is the only site readily accessible for human studies, and it is possible that, at least in some patients, BLyS mRNA levels in circulating leukocytes do not reflect local BLyS production in the secondary lymphoid tissues.

One must also recognize that disease activity in SLE is not solely driven by B cells. Systemic inflammation and SLE flares can be triggered via B cell independent means. Not all SLE patients treated with a B cell depleting course of rituximab experience clinical remission [39], strongly pointing to the importance of non-B cells in disease pathogenesis/maintenance. Conversely, not all pathogenic B cells necessarily require high levels of BLyS to effect their pathogenicity. Murine studies have unequivocally documented B cell subpopulations that do not depend upon BLyS for their survival [40-42]. Although mice completely devoid of BLyS have reduced numbers of mature B cells and harbor reduced levels of immunoglobulin, these reductions are incomplete. Thus, it is possible that some SLE patients harbor pathogenic B cells that are relatively insensitive to BLyS and could drive considerable disease activity even in the context of low endogenous BLyS production. Conversely, patients with high BLyS mRNA levels may be those patients whose disease is strongly driven by BLyS and may be especially helped by BLyS antagonist therapy. Future clinical trials should be able to establish whether the BLyS mRNA levels are good predictors of response to such agents.

## Conclusion

Plasma total immunoglobulin, IgG, and IgA levels and disease activity (as measured by SLEDAI) in SLE patients correlate more closely with peripheral blood leukocyte levels of BLyS mRNA than with plasma levels of BLyS protein. These findings suggest that BLyS mRNA levels better reflect *in vivo *BLyS production than do circulating BLyS protein levels, and may be a useful biomarker in the clinical monitoring of SLE patients. These findings also support the premise that BLyS overexpression not only promotes development of disease but also actively contributes to the ongoing maintenance of disease in SLE patients. This reinforces the rationale underlying clinical trials with BLyS antagonists in SLE.

## Abbreviations

anti-dsDNA = anti-double-stranded DNA; BLyS = B lymphocyte stimulator; bp = base pairs; Ct = threshold cycle; ELISA = enzyme-linked immunosorbent assay; PCR = polymerase chain reaction; RA = rheumatoid arthritis; SLE = systemic lupus erythematosus; SLEDAI = SLE Disease Activity Index.

## Competing interests

TSM and DMH were employees of Human Genome Sciences (HGS) at the time the investigation was conducted. (DMH has since left the company.) WS has received research support from HGS and has served as a consultant to HGS (<$10,000). CEC, ALG, and DN declare that they have no competing interests.

## Authors' contributions

CEC identified and recruited all participants; collected all the blood samples and reviewed all the medical charts; and wrote the initial working draft of this manuscript. ALG developed and performed all the real-time PCR assays and assisted in the interpretation of the results and in writing the final version of the manuscript. TSM performed the plasma BLyS protein and anti-BLyS assays and assisted in the interpretation of the results and in writing the final version of the manuscript. DMH assisted in the design in the study, in the interpretation of the results, and in writing the final version of the manuscript. DN assisted in the design in the study, in the interpretation of the results, and in writing the final version of the manuscript. WS conceived the study, supervised the recruitment of participants, performed the statistical analyses, assisted in the interpretation of the results, and supervised the editing of the manuscript to its final form. All authors read and approved the final manuscript version.

## Figures and Tables

**Table 1:** Characteristics of study populations

Parameter	Normal	RA	SLE	SLE repeat
Number of subjects	30	60	60	37
Age (mean [range]; years)	36.5 (21.2–54.4)	47.3 (19.1–71.7)	39.4 (19.4–66.8)	35.1 (20.4–66.3)
Sex (female/male; *n*)	27/3	52/8	56/4	36/1
				
Race (*n*)				
Asian	11	5	5	2
Black	3	3	6	3
Hispanic	13	51	49	32
White	3	1	0	0
				
Prednisone use				
Number of patients	-	17	50	31
Mean dosage (mg/day)	-	5.5	10.8	6.0
Dosage range (mg/day)	-	2.5–10	2–30	1–30
				
Use of other medications (*n*)				
Cyclophosphamide	-	0	4	2
Azathioprine	-	4	27	15
Mycophenolate mofetil	-	1	9	13
Methotrexate	-	42	2	2
Sulfasalazine	-	17	0	0
Hydroxychloroquine	-	36	53	36
Leflunomide	-	0	1	0
Any TNF antagonist	-	18	0	0

RA, rheumatoid arthritis; SLE, systemic lupus erythematosus; TNF, tumor necrosis factor.
